# The Influence of 3D Technology Integration on Laparoscopic Partial Nephrectomy Practice and Surgical Outcomes

**DOI:** 10.3390/curroncol32060297

**Published:** 2025-05-23

**Authors:** Markos Karavitakis, Nikolaos Grivas, Christos Zabaftis, Filippos Nikitakis, Smaragda Tsela, Ioannis Leotsakos, Ioannis Katafigiotis, Dionysios Mitropoulos

**Affiliations:** 1 2nd Department of Urology, Lefkos Stavros—The Athens Clinic, 11528 Athens, Greece; markoskaravitakis@yahoo.gr (M.K.); zabaftisc@gmail.com (C.Z.); nikitakisf@gmail.com (F.N.); smatsela8@yahoo.gr (S.T.); ioannisdleotsakos@gmail.com (I.L.); katafigiotis.giannis@gmail.com (I.K.); 2 1st Urology Department, National and Kapodistrian University of Athens, 15784 Athens, Greece; dmitrop@med.uoa.gr

**Keywords:** kidney cancer, partial nephrectomy, laparoscopic surgery, three-dimensional technology

## Abstract

Partial nephrectomy (PN) is the standard treatment for renal cell carcinoma (RCC), offering cancer control with renal preservation. Three-dimensional laparoscopy addresses the limitations of traditional two-dimensional systems by enhancing depth perception and accuracy. This study evaluates the impact of 3D laparoscopy on PN for larger and complex tumors. We retrospectively analyzed 200 laparoscopic nephrectomies by a single surgeon between 2020 and 2024, comparing pre-3D and post-3D groups (100 cases each). Key outcomes included the rate of PN, warm ischemia time (WIT), and operative time. The post-3D group demonstrated a significant increase in PN for tumors >4 cm (48% vs. 35%, *p* = 0.028) and high RENAL scores ≥8 (41% vs. 29%, *p* = 0.035). Median WIT was significantly shorter (24 min vs. 29 min, *p* = 0.018 for larger tumors; 26 min vs. 32 min, *p* = 0.022 for high complexity). Total operative time was also reduced (175 min vs. 195 min, *p* = 0.031). Positive surgical margins were lower in the post-3D group (0% vs. 2%), and complication rates were comparable (5% vs. 4%, *p* = 0.712). Three-dimensional laparoscopy significantly improves the feasibility and precision of PN for larger and complex tumors, enhancing outcomes without increasing complications.

## 1. Introduction

Kidney cancer incidence has significantly increased in recent years, with renal cell carcinoma (RCC) representing approximately 90% of cases. Partial nephrectomy (PN) has become the standard of care for managing renal cancer, offering effective oncological control while preserving renal function. This approach notably reduces the risk of chronic kidney disease compared to radical nephrectomy [1]. Compared to open surgery, laparoscopic techniques provide similar oncological outcomes with added benefits, including reduced blood loss, shorter hospital stays, faster recovery, and comparable cancer control [2]. However, traditional laparoscopic surgery using two-dimensional (2D) visualization systems has inherent limitations, such as limited motility of laparoscopic instruments, poor depth of perception, and long learning curve.

In order to handle these critical limitations, new technologies have been developed such as instruments that can mimic surgeon’s hand movement and three-dimensional (3D) visualization systems. Three-dimensional images are created using two different cameras which pass images through an eyeglass that ultimately corresponds to one camera, in order to receive images as one. This technology significantly enhances depth perception, suturing quality, ergonomics, and spatial accuracy, essential for complex procedures such as partial nephrectomy [3]. Despite these advancements, data on the impact of 3D laparoscopy on surgical decision-making remain limited and the 3D laparoscopic system is not yet universally adopted. Questions persist regarding whether 3D systems have influenced the preference for partial nephrectomy over radical nephrectomy or improved oncological outcomes [4]. Regarding surgical outcomes, a recent systematic review has shown the superiority of 3D partial nephrectomy with respect to warm ischemia time, while no differences were found relative to operative time, estimated blood loss, dissecting time, or suturing time [5].

The aim of our study was to evaluate how the implementation of a 3D monitor system modified the practice of partial nephrectomy in a high-volume center, particularly in terms of the adoption of partial nephrectomy for larger and more complex renal tumors. Moreover, differences with 2D laparoscopy with respect to key surgical outcomes were recorded.

## 2. Materials and Methods

### 2.1. Study Design

This retrospective comparative study analyzed 200 consecutive laparoscopic nephrectomy cases performed by a single high-volume surgeon at the same center between 2020 and 2024. The cases were divided into two groups: Group A comprised the 100 cases immediately following the implementation of 3D laparoscopy; Group B comprised the 100 cases prior to the adoption of 3D technology (i.e., those using 2D laparoscopy).

The 3D laparoscopic systems used in this study were the Karl Storz Rubina, which provides advanced depth-perception and high-resolution imaging. This system was utilized consistently for all cases in the post-3D group. To ensure appropriate interpretation of the learning curve and outcome comparisons, it is important to note that the operating surgeon had extensive prior experience with conventional 2D laparoscopic renal surgery. Specifically, before performing the 100 cases included in the 2D group, the surgeon had completed more than 240 laparoscopic partial and radical nephrectomies using 2D systems, including over 160 partial nephrectomies. The final 100 consecutive 2D laparoscopic cases immediately preceding the 3D implementation formed the comparator group in this study. Tumor characteristics, as assessed by size and RENAL nephrometry score, were comparable between the two cohorts, allowing for a robust evaluation of the impact of 3D technology on surgical performance in an otherwise consistent operative environment. Outcomes between the two groups were compared to assess the impact of 3D visualization on the rate of PN, warm ischemia time (WIT), and total operative time.

### 2.2. Methodology

#### 2.2.1. Preoperative Data

Patient demographics and preoperative characteristics included age, gender, tumor location and size as determined by imaging, RENAL nephrometry score, comorbidities, and baseline laboratory values such as hemoglobin and serum creatinine levels.

#### 2.2.2. Operative Data

The collected surgical parameters included warm ischemia time (WIT), estimated blood loss, total operative time, and any additional procedures, such as pelvicalyceal system repair or placement of a double J stent.

#### 2.2.3. Postoperative Data

Postoperative outcomes were evaluated based on the length of hospital stay, changes in hemoglobin and serum creatinine levels at discharge, and complications occurring within 30 days of surgery. Complications were classified using the Clavien–Dindo grading system.

#### 2.2.4. Histopathological Review

A dedicated uropathologist reviewed all histopathological specimens to confirm tumor type and assess margin status.

### 2.3. Statistical Analysis

Data were analyzed using appropriate statistical methods to compare outcomes between the two groups. Continuous variables, such as warm ischemia time, blood loss, and total operative time, were expressed as means with standard deviations and compared using Student’s t-test or the Mann–Whitney U test, depending on normality. Categorical variables, including the rate of partial nephrectomy and complication grades, were analyzed using chi-square or Fisher’s exact test. A *p*-value of <0.05 was considered statistically significant. All analyses were performed using SPSS 22 software.

## 3. Results

### 3.1. Baseline Characteristics

The baseline characteristics of the two groups (pre-3D and post-3D) are shown in Table 1. Both groups demonstrated comparable demographic and clinical parameters, ensuring a balanced comparison.

The mean age and gender distribution were similar between the groups, with no statistically significant differences observed. Preoperative hemoglobin and serum creatinine levels were comparable, with no significant variation between the groups. These findings confirm that the two groups were well-matched, allowing the evaluation of the impact of 3D technology on surgical outcomes without bias from baseline disparities.

### 3.2. Surgical Outcomes

The comparison of surgical outcomes between the pre-3D and post-3D groups is summarized in Table 2. The rate of partial nephrectomies increased significantly in the post-3D group compared to the pre-3D group (68% vs. 57%, *p* = 0.032), suggesting a higher confidence in performing conservative surgeries with the assistance of 3D imaging. While the mean tumor size and RENAL scores were comparable between the two groups (*p* = 0.238 and *p* = 0.187, respectively), the median warm ischemia time (WIT) was significantly reduced in the post-3D group (22 min vs. 27 min, *p* = 0.014). Additionally, the total operative time was also significantly shorter in the post-3D group (68 min vs. 124 min, *p* = 0.042), indicating improved efficiency in the surgical procedure. Although there was a reduction in blood loss in the post-3D group (median 200 mL vs. 250 mL), this difference was not statistically significant (*p* = 0.056). The length of hospital stay was similar between the two groups, as were the rates of complications (*p* = 0.315 and *p* = 0.712, respectively). Importantly, the rate of positive surgical margins was lower in the post-3D group, with no cases observed, compared to 2% in the pre-3D group, although this difference did not reach statistical significance (*p* = 0.104).

### 3.3. Subgroup Analysis for Tumor Size > 4 cm and Higher Complexity (RENAL Score ≥ 8)

A detailed analysis of cases with tumors larger than 4 cm and higher complexity (RENAL score ≥ 8) demonstrated significant benefits from the introduction of 3D technology (Table 3). In the pre-3D period, we had a total of 47 cases with tumors >4 cm, while in the post-3D period, this number was 48. The proportion of partial nephrectomies notably increased from 35% in the pre-3D group to 48% in the post-3D group, highlighting a substantial shift towards more conservative surgeries (*p* = 0.028). Moreover, the median warm ischemia time (WIT) was significantly reduced in the post-3D group (24 min vs. 29 min, *p* = 0.018), as was the total operative time (175 min vs. 195 min, *p* = 0.031).

Similarly, as to cases with a RENAL score ≥ 8, there were 36 cases in the pre-3D group and 41 in the post-3D group. The proportion of partial nephrectomies also showed a significant increase, from 29% in the pre-3D group to 41% in the post-3D group (*p* = 0.035). The introduction of 3D technology was associated with a significant reduction in the median WIT for these high-complexity cases (26 min vs. 32 min, *p* = 0.022), in addition to a substantial decrease in the total operative time (115 min vs. 196 min, *p* < 0.001).

## 4. Discussion

In recent years, kidney preservation during the surgical treatment of renal tumors has become a key objective, as it is associated with better overall survival and reduced risk of chronic kidney disease [1]. This focus has driven an increasing number of reports advocating partial nephrectomy, even for larger and more technically challenging tumors. The adoption of minimally invasive techniques, particularly laparoscopy, has been instrumental in achieving this goal, offering reduced postoperative pain, faster recovery, and oncological outcomes comparable to open surgery [2]. However, its effectiveness has been historically limited by the inherent drawbacks of two-dimensional (2D) visualization, such as poor depth-perception and reduced spatial accuracy. The introduction of three-dimensional (3D) monitoring systems has addressed these challenges, enhancing depth perception and surgical precision, thereby paving the way for significant advancements in laparoscopic techniques [3].

The purpose of our study was to evaluate how the introduction of 3D technology has influenced the practice of laparoscopic kidney surgery. Using a single high-volume surgeon in a single center to minimize variability, the study provides valuable insights into the ways in which 3D technology has influenced clinical practice.

Our study demonstrated that the introduction of 3D laparoscopic technology significantly impacted the practice of kidney surgery. The rate of partial nephrectomies notably increased in the post-3D group, particularly in cases of tumors larger than 4 cm and in cases with higher complexity as indicated by RENAL nephrometry scores. These findings suggest that the enhanced visualization provided by 3D systems enables surgeons to tackle more challenging cases laparoscopically.

In addition, the implementation of 3D technology was associated with reductions in both warm ischemia time and total operative time across all subcategories, including cases involving larger tumors and those with higher complexity. A reduction in warm ischemia time was found also in the meta-analysis of Dirie et al., while no difference was shown as to operative time [5]. However, a meta-analysis by Sánchez-Margallo et al. demonstrated a reduction in operative time when 3D laparoscopic imaging systems were used for various urological procedures, particularly those requiring intracorporeal ligatures and sutures, such as partial nephrectomy. This reduction was accompanied by decreased blood loss and reduced stress and workload for the surgeon during the procedure [6]. A study by Tokas et al. (2021) [7] demonstrated also that 3D visualization enhanced depth perception and spatial awareness during laparoscopic PN for renal tumors, resulting in shorter warm ischemia times and improved surgical precision. These advantages allowed surgeons to confidently manage larger and more complex tumors laparoscopically, further advancing nephron-sparing approaches [7].

Similar to the results of the meta-analysis of Dirie et al. [5], our study has found no differences in the complication rates and hospital stays between the 2D and 3D groups, demonstrating that the safety profile of laparoscopic surgery was maintained even when managing more complex cases. Furthermore, there was a trend toward fewer positive surgical margins in the post-3D group, although this was not statistically significant.

Gupta et al. (2019) [8] evaluated the use of “no clamp” or zero ischemia techniques in 3D laparoscopic PN. Their findings highlighted the precision enabled by 3D systems, which minimized vascular clamping and preserved renal function, even in challenging tumor cases. The study emphasized the utility of 3D technology in facilitating advanced surgical techniques while reducing ischemic stress on the kidney [8]. Furthermore, Cicione et al. assessed the impact of 3D technology on novice laparoscopic surgeons using a porcine kidney model. Their quantitative analysis revealed faster skill acquisition, better accuracy, and reduced error rates compared to 2D systems, showcasing the broader educational benefits of 3D laparoscopy for both novice and experienced surgeons [9].

These findings are consistent with the outcomes observed in our study, in which the adoption of 3D laparoscopy led to an increased rate of partial nephrectomies for larger tumors (>4 cm) and more complex cases (RENAL score ≥8). Additionally, 3D systems reduced warm ischemia time and total operative duration across all tumor subcategories, while maintaining comparable complication rates and safety profiles. As corroborated by systematic reviews and meta-analyses, particularly with respect to urological surgeries and other surgical specialties, 3D laparoscopy consistently improves perioperative and oncological outcomes while expanding the indications for minimally invasive surgery [10,11,12]. The advantages of 3D laparoscopy in surgical outcomes extend to other aspects of kidney surgery, for example, in the treatment of benign nonfunctioning kidneys with 3D laparoscopic nephrectomy [13]. In another study, which investigated the feasibility of bench surgery combined with autotransplantation following 3D laparoscopic nephrectomy for the treatment of complex kidney tumors, 3D technology facilitated safe and atraumatic dissection of the renal pedicle vessels, an important surgical step also relevant in laparoscopic partial nephrectomy [14].

The cumulative evidence highlights the transformative role of 3D technology in improving surgical precision, facilitating complex procedures, and enhancing training. As seen across various studies and surgical disciplines, 3D laparoscopy represents a significant step forward in minimally invasive techniques, providing benefits that extend from patient outcomes to surgeon ergonomics and education [15,16]. This growing body of evidence underscores the importance of integrating 3D technology into laparoscopic surgery to advance patient care and surgical capabilities.

Our study represents a significant contribution to the literature, as it is the first to demonstrate how the implementation of 3D laparoscopic systems has fundamentally altered clinical practice in kidney surgery. By comparing two large, consecutive cohorts managed by the same high-volume surgeon, we minimized confounding variables related to surgical expertise and institutional differences, ensuring the reliability of our findings. The increased rate of partial nephrectomies for larger and more complex tumors, coupled with the reduction in warm ischemia time and total operative time, highlights the transformative impact of 3D technology in enabling more precise and efficient minimally invasive surgery.

In recent years, robotic-assisted surgery has been widely used in a variety of urological surgical procedures, demonstrating noticeable improvements in perioperative surgical outcomes, especially regarding nephron-sparing surgery for kidney tumors [17,18]. However, robotic platforms are still not available in many surgical centers around the world, in which laparoscopy remains the sole option for minimally invasive surgery [19]. Among other ergonomic advantages, one key characteristic of robotic platforms is 3D visualization, which significantly contributes to the precision of robotic-assisted surgery. Nevertheless, as noted above, this is also a feature that can be implemented in a laparoscopic setting, and it can assist in achieving surgical accuracy comparable to those of the robotic platforms, further improving the efficacy and safety of laparoscopic kidney surgery. Thus, 3D technology enables advanced laparoscopic nephron-sparing surgery in centers where robotic-assisted surgery is not yet accessible, and it can broaden the access to high-quality minimally invasive kidney surgery, also in the context of low-resource countries.

However, the study has some limitations. Its retrospective design, while robust in controlling for certain variables, may introduce inherent biases in data collection and analysis. Additionally, our findings are based on the experience of a single surgeon with a high (and increasing) level of experience, which, while eliminating inter-surgeon variability, may limit the generalizability of the results to other clinical settings or less-experienced surgeons. Moreover, while the complication rates were comparable between the two groups, a larger sample size may be necessary to detect subtle differences in safety profiles. Finally, long-term functional outcomes will be useful for the assessment of renal function preservation. Future research should involve multicenter clinical trials with extended postoperative follow-up to validate our findings.

Despite these limitations, this study provides compelling evidence of the clinical benefits of 3D technology and serves as a foundation for future research. Evolving studies have shown that 3D models in laparoscopic partial nephrectomy, combined with artificial intelligence-based augmented reality, have facilitated intraoperative real-time navigation, creating a surgical roadmap that improves operative efficiency [20]. By demonstrating that 3D systems not only improve surgical outcomes but also expand the scope of laparoscopic kidney surgery, our work underscores the need for widespread adoption of this technology in modern urological practice.

## 5. Conclusions

In conclusion, the integration of 3D laparoscopic technology represents a significant advancement in the field of minimally invasive urological surgery. By enhancing depth perception and spatial accuracy, 3D systems have transformed the capabilities of laparoscopic procedures, allowing for more precise and confident management of complex renal tumors. This evolution in visualization technology not only supports a more conservative surgical approach but also aligns with the growing emphasis on nephron-sparing techniques. As 3D laparoscopy continues to expand its role in clinical practice, further research and wider adoption may pave the way for even greater improvements in surgical outcomes and patient care.

## Figures and Tables

**Table 1:** Baseline characteristics of the study population.

Variable	Pre-3D Group (Group A, n = 100)	Post-3D Group (Group B, n = 100)	*p*-Value
Age (mean ± SD)	56.2 ± 12.8	55.7 ± 13.1	0.764
Gender (M/F)	63/37	60/40	0.562
Tumor laterality (right/left)	48/52	50/50	0.832
Tumor size (cm) (mean ± SD)	4.3 ± 1.2	4.1 ± 1.3	0.472
RENAL nephrometry score (mean ± SD)	6.4 ± 1.0	6.3 ± 1.1	0.635
Baseline hemoglobin (mean ± SD)	13.5 ± 1.4	13.6 ± 1.3	0.721
Baseline creatinine (mean ± SD)	0.92 ± 0.3	0.91 ± 0.3	0.788

SD, standard deviation; M, males; F, females.

**Table 2:** Comparisons of surgical outcomes.

Variable	Pre-3D Group (Group A, n = 100)	Post-3D Group (Group B, n = 100)	*p*-Value
Partial/Radical nephrectomy	57/43	68/32	0.032
Tumor size (cm) (mean ± SD)	4.7 ± 1.9	4.5 ± 1.8	0.238
RENAL score (mean ± SD)	6.3 ± 1.2	6.5 ± 1.4	0.187
WIT (min) (median [IQR])	27 [23–31]	22 [18–27]	0.014
Total operative time (min) (median [IQR])	124 [80–154]	68 [47–75]	0.042
Blood loss (mL) (median [IQR])	250 [200–350]	200 [150–300]	0.056
Hospital stay (days) (median [IQR])	2 [2–4]	2 [2–3]	0.315
Complications (≥CD II)	4%	5%	0.712
Positive margins (Y/N)	2/98	0/100	0.104

SD, standard deviation; WIT, warm ischemia time; IQR, interquartile range; CD, Clavien–Dindo classification grade; Y, yes; N, no.

**Table 3:** Subgroup analysis—tumors > 4 cm and higher complexity (RENAL score ≥ 8).

Subgroup Analysis	Variable	Pre-3D Group (Group A, n = 100)	Post-3D Group (Group B, n = 100)	*p*-Value
Tumors > 4 cm	Partial/Radical nephrectomy	16/31	23/25	0.028
	WIT (min) (median [IQR])	29 [25–33]	24 [20–28]	0.018
	Total operative time (min) (median [IQR])	195 [170–220]	175 [150–200]	0.031
Higher Complexity (RENAL ≥ 8)	Partial/Radical nephrectomy	10/26	17/24	0.035
	WIT (min) (median [IQR])	32 [28–36]	26 [22–30]	0.022
	Total operative time (min) (median [IQR])	196 [190–230]	115 [110–120]	<0.001

WIT, warm ischemia time; IQR, interquartile range.

## Data Availability

The data shown in this study are available from the corresponding author upon reasonable request.
